# Monitoring the Environmental Aging of Nanomaterials: An Opportunity for Mesocosm Testing?

**DOI:** 10.3390/ma12152447

**Published:** 2019-07-31

**Authors:** Armand Masion, Mélanie Auffan, Jérôme Rose

**Affiliations:** 1 CNRS, Aix Marseille Université., IRD, INRA, Coll France, CEREGE; Europole Arbois, BP 80, 13545 Aix en Provence, France; 2 Labex SERENADE, Europole Arbois, 13545 Aix en Provence, France; 3 Civil and Environmental Engineering, Duke university, Durham, NC 27708, USA

**Keywords:** nanomaterial, environmental aging, mesocosm, material life-time

## Abstract

Traditional aging protocols typically examine only the effects of a limited number of stresses, and relatively harsh conditions may trigger degradation mechanisms that are not observed in actual situations. Environmental aging is, in essence, the complex interaction of multiple mechanical, physicochemical and biological stresses. As yet, there is no (pre)standardized procedure that addresses this issue in a satisfactory manner. Mesocosm experiments can be designed to specifically cover the aging of nanomaterials while characterizing the associated exposure and hazard. The scenario of exposure and the life time of the nanomaterial appear as the predominant factors in the design of the experiment, and appropriate precautions need to be taken. This should the subject of guidance that may be divided into product/application categories.

## 1. The Importance of the Aging Factor

There are still major knowledge gaps in the risk assessment of nanomaterials, especially in the post-production stages of their life cycle. However, in most cases, the use and end-of-life phases are by far the longest periods, and this extended duration is a potential concern with regard to exposure to nanomaterials, as well as associated hazards. Apart from a few exceptions, nanomaterials with the desired properties are not used “as is”, but are embedded in the final product after several formulation steps (e.g., encapsulation, attachment/embedding into a matrix, etc.) for targeted functionality, better product safety, or simply ease of use. As a consequence, the pristine/isolated nanoparticle is no longer the only compound that needs to be considered in risk assessment. The nature and the structure of the bearing matrix control the exposure to the nanomaterial and the associated hazard. In other words, the entire nano-enabled product and its evolution with time, i.e., aging, become determining factors in the risk assessment.

While a newly manufactured product should not be a concern for the consumer or the environment, aging is likely to change the mobility and the speciation of embedded nanomaterials, and may cause potential adverse effects towards the consumer and/or the environment. As a consequence, attention needs to be paid to the degradation of the matrix, shell(s) and coating(s) surrounding the nanomaterial in order to avoid its release in a form that is, or might become, hazardous. It is noteworthy that understanding the (bio)physicochemical mechanisms of matrix degradation leading to nanomaterial release is challenging and requires the use of powerful characterization tools [1,2,3]. Therefore, even if release is a direct consequence of the degradation of the matrix, aging is often studied via the quantification of the release with simple metrics. 

The extent of the phenomena is a function of the stability of the final material, i.e., its resistance to the degradation by several factors (mechanical, physicochemical, biological). At this point, there is a correlation to be made between material stability and the expected life (usage) time of the product. Indeed, a product expected to last a decade or longer (e.g., self-cleaning glass, paint) is designed with durability requirements that differ greatly from those applied to products to be used for only a few hours (e.g., cosmetics). In a first approximation, these differences will also influence the behavior at the end-of-life stage. 

## 2. Traditional Approaches for Characterizing Aging 

**Figure 1:**
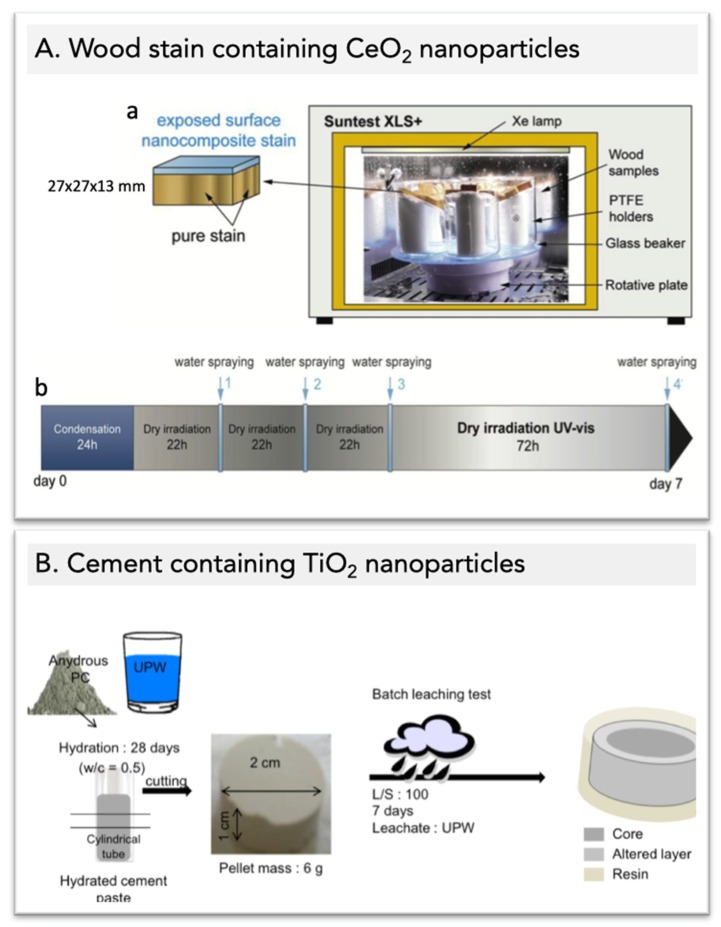
Experimental setup and procedure for the aging of two nano-enabled products with contrasting matrix (from [1,2]). (**A**) Experimental setup and procedure for weathering in climate chamber of wood stain containing CeO_2_ nanoparticles. (**a**) Experimental setup inside the climate chamber (Suntest XLS+). (**b**) Weekly weathering cycle with gray areas corresponding to dry irradiation (UVvis) phases and 20 min spraying events (MilliQ-water) in light blue. (**B**) Experimental setup and procedure for the static leaching test of cement containing TiO_2_ nanoparticles. Batch tests were performed during seven days at liquid-to-solid-weight ratio (L/S) of 100 with ultra-pure water.

Differences in product design strategy according to the expected life time have obvious consequences on how aging is approached from a risk assessment perspective. The aging of products with short life times can be monitored throughout the entire use phase of the material. This means that materials with a real degradation level are available for further (i.e., environmental) testing (see e.g., [4]). As mentioned above, it is likely that the necessary duration for relevant end-of-life monitoring will be reasonably short. Of course, what constitutes “reasonably short” needs to be defined. From a purely scientific point of view, a “reasonably short” duration could easily extend to several months (determination of kinetics, mechanistic studies, validation of predictive models, etc.). Such a duration is obviously not applicable to regulatory purposes; the logistics, the manpower and the analytical requirements are far too resource-intensive. In this context, shorter and more cost-effective tests will be given preference, as exemplified in the aging of wood stain and cement in Figure 1. Then, the duration of the full aging procedure would be expressed in weeks, rather than in months. Wherever the line is drawn for “reasonably short” depending of the context, time is not a limiting factor when examining the aging of short-lived nanomaterials.

The issue is, of course, different for materials intended to be used over long periods of time. Here, real-time monitoring becomes impossible, although there are some isolated reports on the characterization of the initial stages of long-term aging of nano-enabled products (e.g., [5]). The solution is to simulate aging to gain some insight into the nature and quantity of the material released during the actual life time of the product [6]. The strategy consists in applying a large stress to the material over a short period of time (with respect to the actual use) to accelerate/exaggerate the degradation process. It is indisputable that this strategy is flawed, since it is likely that applying a large stress will trigger release mechanisms that do not occur during real aging. However, simulated aging is a necessary operational compromise (and is quantifiable with simple metrics). Also, since harsher aging conditions intuitively cause more damage to the product, the measured release can be considered as a worst-case scenario. From a risk assessment standpoint, one can see the benefits of overestimating the released quantity. Nevertheless, in terms of the nature and structure of the released material, the information obtained with accelerated aging is probably less relevant. 

Aging methods typically investigate the effect of one or a few mechanical and/or physicochemical stresses. For example, in the case of cement, these stresses include abrasion, drilling, heat, humidity, acidity, etc. [7]. A number of these tests are in the form of international standards or guidelines; as an example, there is a series of aging tests available for paints [8,9,10]. These procedures are not necessarily nano-specific, but they can often be applied at the nanoscale with little or no adaptation [11]. These reference methods are very important in determining product durability and safety. For this purpose, it is necessary to strictly adhere to the experimental protocol. This is facilitated by keeping the systems simple with a limited number of parameters to control. Indeed, there is little or no room for uncertainty for methods used as industry standards; reproducibility, accuracy and ease of implementation are key characteristics of this type of tests.

In this context, it is not surprising that this still holds true for environmental aging, an area inherently linked to variability. However, when taking cement and paint as examples of materials with a long use period, the “environmental aging” tests are basically limited to exposing the materials to water immersion/sprinkling and artificial sunlight cycles [9,10]. In this case, accelerated aging might no longer be the worst-case scenario for release because, even from a purely abiotic point of view, most parameters are ignored. Indeed, basic environmental parameters such as salt composition and concentration, presence and nature of organics, and redox cycling are typically not accounted for in the testing procedures. Adding biological activity, which may greatly affect material degradation, increases the system complexity to a level that is not addressed by current standardized environmental aging methods. As a result, it is unclear to which extent the nature and also the quantity of released materials reflect actual situations.

## 3. Mesocosm as an Aging Method 

Mesocosm testing is an increasingly popular alternative to traditional procedures for examining the environmental risks of nanomaterials, because (i) by design, it accounts for system complexity to a level that no other single test does, and (ii) multiple exposure and hazard parameters/end points can be characterized in a single experiment, based on real time monitoring (e.g., multi-parameter probes) and off-line analyses [12,13,14,15,16,17,18,19]. The general strategy behind mesocosm testing is in sharp contrast with detailed parameter/end-point determination; while strict control of every aspect of the procedure is at the core of a traditional standard, mesocosms are expected to evolve freely after their set-up and stabilization period [13] for durations of at least several weeks, and up to a year or longer in some cases [18,20]. The obvious benefit of this testing method is an unparalleled environmental relevance (assuming adequate scenarios of contamination) compared to any other standard or guidance document. On the other hand, this relevance, which to a large extent rests on low concentrations of contaminants, causes analytical challenges, and thus limits parameter determination.

Research on the release of materials from nanoproducts is growing, and the next necessary step is to investigate the behavior and effects of these released materials in the environment and on humans [21]. Herein, we propose using mesocosms as a testing strategy making it possible to study, under relevant environmental conditions, both material aging and environmental impacts. Despite increasing popularity, the potential of mesocosm testing has never been evaluated beyond examining hazard and exposure in re-created ecosystems, yielding, in particular, time-resolved (bio)distribution of the nanomaterials and the associated biological response. As an example, the distribution of Ce in an aquatic mesocosm, expressed as a percentage of the introduced material, was about 1% in the water column, 99% at the surface of the sediment and less than 0.1% in *P. Corneus* in the case of a single dosing contamination scenario, whereas for multiple dosing, these proportions were 89%, 11% and <0.1%, respectively [15]. While it is acknowledged that the combination of multiple bio-physicochemical stresses may cause transformations of the nanomaterial during the experiment, mesocosm testing is only rarely designed to specifically cause or address aging [12,22]. 

The duration of a typical mesocosm experiment is quite compatible with the aging of short-lived nano-enabled products such as cosmetics. In such cases, the entire use phase of the product and a significant portion of its end-of-life stage fit in a single experiment. For products with a long life time, the flexibility of mesocosm testing in terms of duration permits the investigation of the initial stages of aging in a meaningful manner, i.e., over several months, or longer if necessary, although this may not be suitable for regulatory purposes. To address more advanced stages of degradation, it is of course possible to use pre-aged materials for the contamination phase of the mesocosm experiment. In previous studies, a methodology has been developed (such as the use of fragmented products) so as to speed up the degradation of long-lived materials, notably by increasing the surface area at the product/environment interface while keeping the material identical to the real product [12,21]. 

At this point, attention needs be drawn to the fact that mesocosm testing should not be considered a quantitative method for generating environmentally aged materials. Indeed, to be efficient, this would imply introducing high concentrations of material into the mesocosm, which might trigger ecosystem responses that would not occur at actual/predicted contamination levels. Instead, this method monitors the effects of nanomaterial aging as a bio-physicochemical dynamic process. Taking into consideration the possible chemical/structural evolution of the added contaminant increases the complexity of the system, and mesocosm testing is one of the rare methods capable of account for this complexity.

## 4. Working towards Guidance 

Because system parameters are allowed to evolve freely, and because of the analytical challenges arising from low contaminant concentrations, mesocosm testing might be considered unfit for standardization in its traditional sense. In addition, as a matter of fact, it appears to be difficult to harness the versatility of the mesocosm methodology and possible variability of the system responses into a single standard. In fact, there is no single mesocosm experiment covering all possible situations (e.g., aquatic vs. terrestrial, continental vs. marine, etc.). With appropriate guidance, mesocosm testing can address typical environmental situations as well as specific/unusual environmental conditions. Mesocosm testing is generic because of its adaptability, and not because of a unique experimental protocol [13]. However earlier work established a proof-of-concept for the usability of the mesocosm methodology in a Standard Operating Procedure (SOP) [23]. This was accompanied by a clear trend to define a set of criteria for performing meaningful mesocosm experiments with improved comparability of the results. Examples of key parameters to include in a SOP have been given elsewhere (e.g., pH, temperature, dissolved O_2_, etc.).

**Figure 2:**
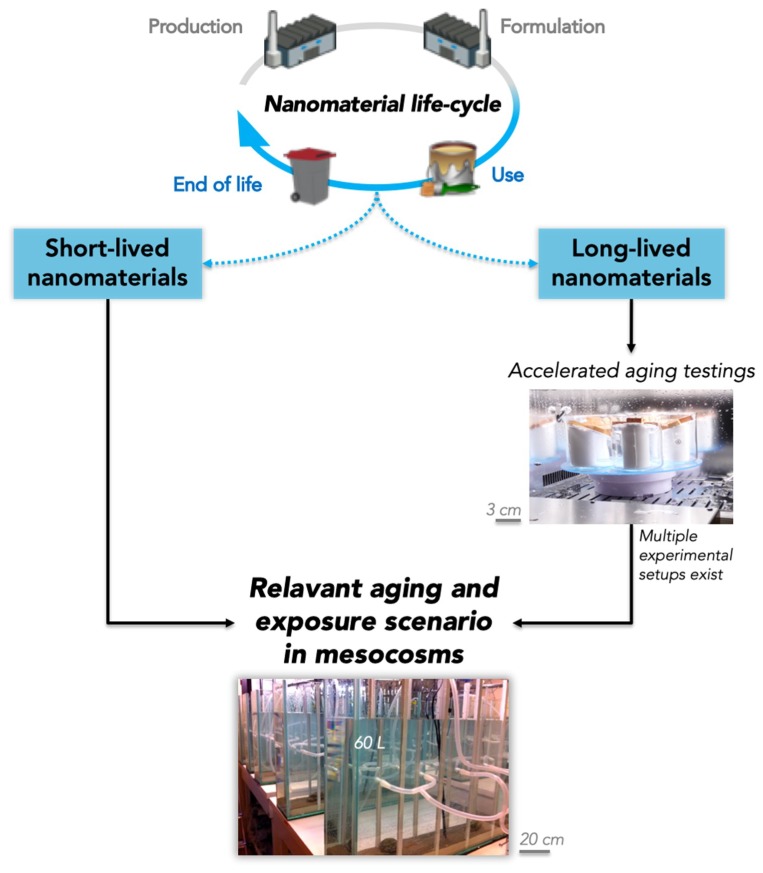
Application type-driven strategy to characterize environmental aging of nanomaterials. Short-lived materials can be aged in the mescocosms; long-lived materials require sample preparation prior to their introduction in the mesocosm.

The determining step for every mesocosm experiment is the selection of the contamination scenario, and this is particularly true when it is dedicated to addressing aging. The versatility of mesocosm testing makes it possible to deliberately focus on aging and the characterization of the associated risk. In this context, an efficient way to define the scenario is to adapt it with respect to the application type of the product (Figure 2). The application type will define the duration, or at least the duration range, of the mesocosm test, and consequently how much of the global aging process can be covered in a single experiment. For example, for short-lived materials, it is possible to set the duration to include the use-phase and possibly the end-of-life phase as well.

For materials with a longer life time, a mesocosm experiment aiming at covering aging can only address a given stage of the process (e.g., initial end-of-life stage) and may have an extended duration. The use of pre-aging procedures becomes a necessary step in generating material with the desired aging/degradation level. 

The duration can be defined by what is considered reasonable/possible in terms of logistics and cost, especially from a standardization/regulatory perspective, but it is necessarily limited by the typical life time of the biota introduced into the mesocosms.

To be environmentally relevant, the contaminant dose should be on the order of actual or predicted contaminant concentrations. The choice between a pulse or chronic contamination scenario is not a logistical issue for short experiments, but for longer durations, applying a low dose over several months (or longer) might become impractical in terms of delivering a constant contaminant dose, and, in the case of pre-aged nanomaterials, prevent their further degradation prior to introduction into the mesocosm. 

As indicated above, mesocosm-driven aging is not the best candidate for a rigid and detailed testing protocol, since it will be governed by the scenario of exposure. Nevertheless, there is a need for a sound guidance to ensure the environmental relevance of the experiment, as well as the usefulness and comparability of the results. Such a document will need to address specific precautions to take when the mesocosm test is meant to cover aging. In this respect, grouping the recommendations regarding experiment design by application type (e.g., cosmetics, paints and stains, etc.) might be an interesting feature. Indeed, nanomaterials in a particular application type should be tested with similar exposure scenarios, given the similarity of the life cycle duration and the likelihood and extent of alteration in order to produce comparable results. This categorization would be a contribution to current grouping efforts (e.g., the EU H2020 program GRACIOUS), and may serve as added guidance in the emerging regulatory process.
